# Genome-wide microarray analysis of TGFβ signaling in the *Drosophila *brain

**DOI:** 10.1186/1471-213X-4-14

**Published:** 2004-10-08

**Authors:** Maocheng Yang, Don Nelson, Yoko Funakoshi, Richard W Padgett

**Affiliations:** 1 Waksman Institute, Department of Molecular Biology and Biochemistry, Cancer Institute of New Jersey, Rutgers University, Piscataway, NJ 08854-8020, USA; 2 Biological Sciences, University of the Cariboo, Kamloops, Britsh Columbia, (V2C 5N3), Canada

## Abstract

**Background:**

Members of TGFβ superfamily are found to play important roles in many cellular processes, such as proliferation, differentiation, development, apoptosis, and cancer. In *Drosophila*, there are seven ligands that function through combinations of three type I receptors and two type II receptors. These signals can be roughly grouped into two major TGFβ pathways, the *dpp*/BMP and activin pathways, which signal primarily through *thick veins (tkv) *and *baboon (babo)*. Few downstream targets are known for either pathway, especially targets expressed in the *Drosophila *brain.

**Results:**

*tkv *and *babo *both affect the growth of tissues, but have varying effects on patterning. We have identified targets for the *tkv *and *babo *pathways by employing microarray techniques using activated forms of the receptors expressed in the brain. In these experiments, we compare the similarities of target genes of these two pathways in the brain. About 500 of 13,500 examined genes changed expression at 95% confidence level (P < 0.05). Twenty-seven genes are co-regulated 1.5 fold by both the *tkv *and *babo *pathways. These regulated genes cluster into various functional groups such as DNA/RNA binding, signal transducers, enzymes, transcription regulators, and neuronal regulators. RNAi knockdown experiments of homologs of several of these genes show abnormal growth regulation, suggesting these genes may execute the growth properties of TGFβ.

**Conclusions:**

Our genomic-wide microarray analysis has revealed common targets for the *tkv *and *babo *pathways and provided new insights into downstream effectors of two distinct TGFβ like pathways. Many of these genes are novel and several genes are implicated in growth control. Among the genes regulated by both pathways is *ultraspiracle*, which further connects TGFβ with neuronal remodeling.

## Background

TGFβ pathways are conserved between primitive animals, such as sponges and sea anemone [1,2] and vertebrates, thus representing an ancient signal transduction pathway. In both vertebrates and invertebrates, TGFβ family members play fundamental roles in proliferation, pattern formation, apoptosis, and specification of cell fate. Mutations of various TGFβ signaling components are associated with human diseases including cancer [3].

In recent years, the core signaling components of the TGFβ pathways have been elucidated by a combination of genetics and biochemical approaches. Unique to these signaling pathways are transmembrane receptor serine-threonine kinases that are novel in animals. Signaling is initiated when dimeric ligands bind to the type I receptor or a complex of the type I and type II receptors. The type II receptor phosphorylates the type I receptor, which renders it active. R-Smads are phosphorylated by the type I receptor, the complex with a co-Smad, and translocate to the nucleus. Smads bind DNA promoter elements weakly and require co-factors for efficient regulation of target genes.

In *Drosophila*, seven ligands have been identified from the genomic sequence [4-6]. These ligands act through a receptor complex comprised of heterodimeric combinations of type I and type II receptors. Three type I receptors, *thick veins (tkv)*, *saxophone*, *baboon (babo) *and two type II receptors, *punt and wishful thinking (wit)*, interact with either of two R-Smads, *mothers against dpp (mad) *or *dSmad2 *[7-12]. Although different heteromeric combinations of receptors exist, in general, *tkv *transmits a *dpp*/BMP signal through *mad*, and *babo *transmits an activin signal through *dSmad2*.

The *dpp *and activin pathways have known functions in the brain, although our understanding of it role is rudimentary. *dpp *is expressed in two areas adjacent to the outer proliferation center (OPC), where it modulates wingless expression [13]. To acquire the adult pattern of projections, extensive remodeling occurs in neurons of the larval neural circuits during metamorphosis [14]. Proper neuronal remodeling is important for transformation of the larval mushroom bodies (MBs) to the adult MBs [15,16]. *babo *and dSmad2 (activin pathway components) are involved in neuronal remodeling, which occurs in the larval-pupal transition [17]. One target of the activin pathway identified in these studies is a subunit of the ecdysone receptor, EcR-B. Neuronal remodeling is essential for brain development in most animals and this result raises the question of possible conservation of neuronal targets in vertebrates.

In spite of intense study using classical genetic approaches and biochemical methods, very few targets of the pathway have been identified. A better understanding of the growth and patterning properties of the pathway require a more complete list of target genes. Using activated receptors (*tkv *and *babo*), we have used microarray technology to identify common targets of the BMP and activin pathways in the *Drosophila *brain.

## Results and discussion

### Identifying targets of *babo *and *tkv *in *Drosophila *brains

Few targets of *tkv *signaling and even fewer targets of *babo *signaling are known in *Drosophila*. Though multiple ligands and type II receptors may interact with these type I receptors, the use of ligand/receptor combinations is not yet established with certainty. However, in a simplified view, *tkv *and *babo *send *dpp *(BMP) and activin signals. These pathways and receptors are conserved through evolution, but few downstream targets are known for these pathways in any organism. To learn more about the growth regulatory and pattering properties of these signals in the fly brain, we used microarray technology to identify downstream targets. In these experiments, Affymetrix™ chips containing the entire protein coding capacity of the *Drosophila *genome (about 13,500 genes) were screened. Genomic-wide microarray analysis allows us to examine similarities and differences between two signaling pathways in a tissue where both are known to function.

Constitutively active forms of the receptors were made by single amino acid substitutions [18], rendering them active in the absence of ligand. Transformants were generated which could be transcriptionally expressed using the *heat shock *GAL4 driver (*hs*-GAL4) [19,20]. To assay for the best induction protocol, animals were heat shocked and monitored for the presence of a UAS-*gfp *reporter. Since additional time is required for the induction of downstream signaling targets *versus *the time required for appearance of the GFP reporter, we collected RNA samples from third instar larvae in a broad time period roughly 30 minutes after the peak of GFP expression. Data resulting from induced ectopic expression of *tkv*, and *babo *were compared with each other and to the control (UAS-*gfp; hs*-GAL4).

**Figure 1:**
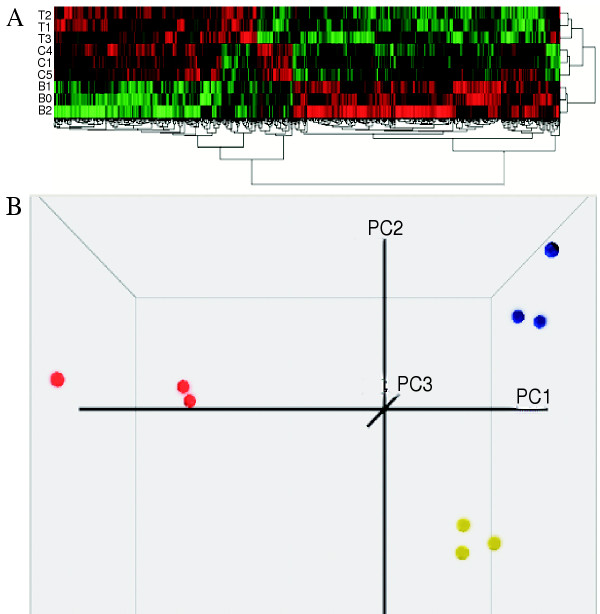
Clustering of microarray data. A. Agglomerative Hierarchical Clustering of microarray data (P < 0.05, Group T, C, B represent individual samples with ectopic expression of *tkv*, control, and *babo *respectively); B. Principal Component Analysis (PCA) of microarray data (P < 0.05, Spheres in red, blue and yellow represent individual samples with ecotopic expression of *babo*, *tkv*, and control respectively).

Three independent replicates for each treatment were generated. Hierarchical clustering (Fig 1A) and Principal Component Analysis (Fig 1B) indicated that the microarray data is highly reproducible. Only transcripts that show an expression level above 1.5 fold change at significance values of P < 0.05 (Anova) were considered to be differentially expressed. These experiments identified genes that are either regulated by both *tkv *and *babo *pathways or by one of the pathways only.

**Figure 2:**
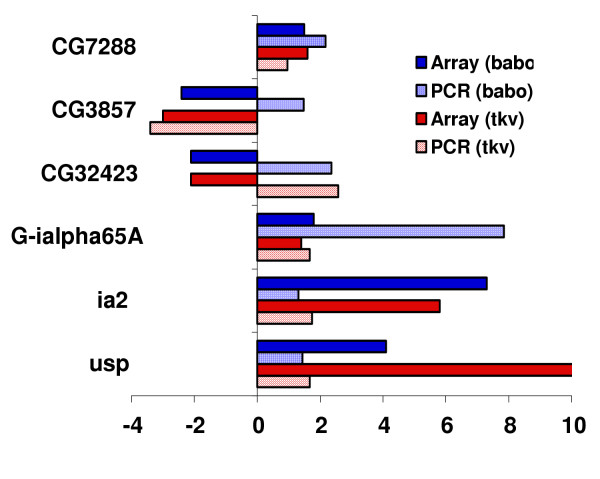
Validation of microarray data by real time RT-PCR. The X-axis indicates fold changes (FC) of gene expression levels between *tkv/babo *ectopic expression and control (Positive values indicate that the relative expression level of a gene is increased and negative values indicate a decrease). Array /PCR (*tkv*, *babo*) represents the fold changes of transcripts with ectopic expression of *tkv*, *babo *in microarray /Real time PCR respectively.

To verify the differential expression levels in response to ectopic expression of *tkv *and *babo *on microarrays, semi-quantitative real-time RT-PCR was performed on selected genes. Six among the 27 genes were picked for validation. Real time RT-PCR showed similar results similar to the microarray results for four of the six genes (Fig 2). These PCR results are consistent with those of other reported microarray experiments [21,22]. This data, with the reproducibility of the individual samples analyzed, establish the validity of our microarray data and provide a comparison of two signaling pathways in the *Drosophila *brain.

### Overview of gene expression following ectopic expression of *babo *and *tkv*

**Figure 3:**
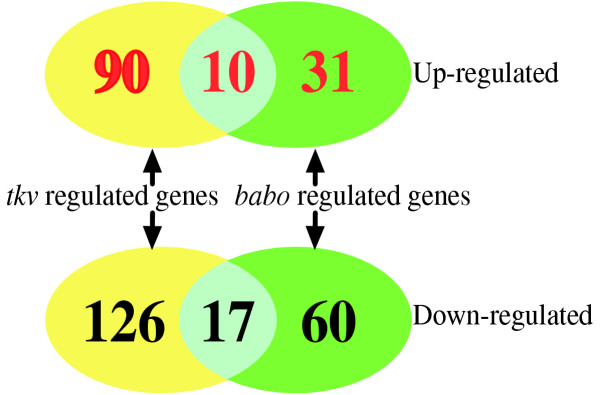
Distribution of differential regulated genes by ecotopic expression of *tkv *and *babo*. Upon ectopic expression of *tkv*, 60 transcripts are downregulated and 31 are upregulated. Similarly, at ectopic expression of *babo*, we detected 216 genes with differential expression values in brain tissues and expression levels of 126 transcripts are decreased and 90 transcripts are increased. There are 27 genes coregulated at ectopic expression of both *babo *and *tkv*. 17 of these transcripts are downregulated and 10 of transcripts are upregulated.

Upon ectopic expression of *tkv*, 91 transcripts are detected with differential expression values in brain tissues when compared with the control (Fig 3). This corresponds to about 0.7% of the transcripts on the array. More transcripts are down regulated (n = 60) than up regulated (n = 31) in abundance levels, indicating that ectopic expression of *tkv *causes both repression and activation of downstream genes. Induction of activated *babo *results in 216 genes with differential expression values in brain tissues. Interestingly, expression levels of more transcripts are decreased (n = 126) than increased (n = 90) (Fig 3). This corresponds to about 1.6% of the transcripts on the array. Most importantly, there are 27 genes co-regulated by induction of both *babo *and *tkv *– 17 of these transcripts are down regulated and 10 of them are up regulated (Fig. 3).

### Role for TGFβ signaling in neuronal remodeling

**Figure 4:**
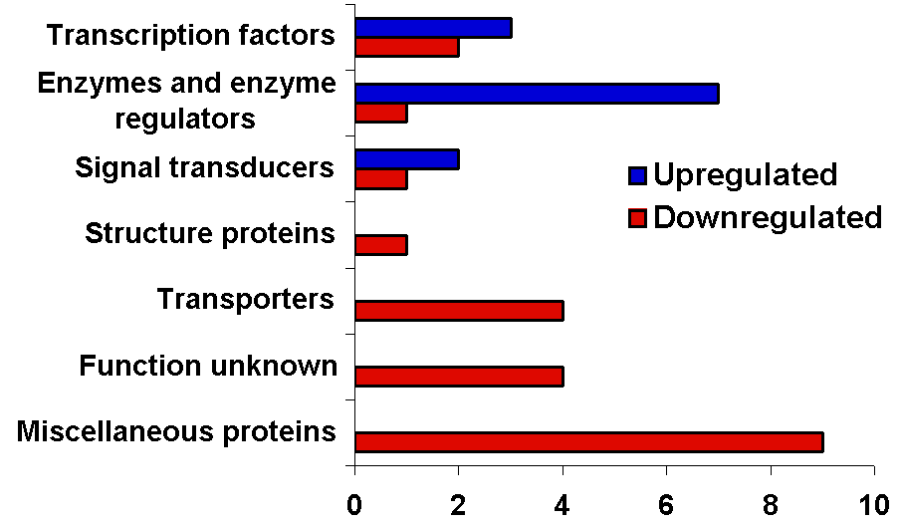
Gene ontology of coregulated genes by both DPP and activin signaling pathways. The X-axis indicates the number of genes in each group.

The fact that both DPP and activin signaling pathways share some common features in differentiation and growth control in various tissues suggests that both pathways might share some downstream target genes. Microarray experiments identified 27 genes (Table 1) co-regulated by the induced expression of both *tkv *and *babo*. Among these 27 co-regulated transcripts, there are transcription factors, enzymes, transporters, signal transducers, miscellaneous proteins and four unknown genes (Fig 4). The transcription factor *ultraspiracle *(*usp*) gene has the highest expression level increase (8.1-fold for *babo*, 27.3-fold for *tkv*), which is a subunit of a nuclear receptor [15]. USP forms a heterodimer with the nuclear ecdysone receptor (EcR) and participates in neuronal remodeling [15,23].

**Table 1:** Changes in transcript levels of the coregulated genes by both *tkv *and *babo *pathways after ectopic expression of *tkv *and *babo*. (*FC represents the fold changes in gene expression levels between *tkv/babo *ectopic expression and control. Positive values indicate that the relative expression level of a gene is increased (upregulation) and negative values indicate a decrease (downregulation)).

Gene/synonym	Signal		FC*		Molecular function	P
			
	*babo*	*tkv*	*babo*	*tkv*		
**Transcription factors**						
*usp*	41	137	8.1	27.3	transcription factor, DNA binding, ligand-dependent nuclear receptor, ecdysteroid hormone receptor	0.0001
CG7839	36	34	1.7	1.6	transcription factor	0.0007
TfIIFβ	208	249	1.2	1.5	RNA polymerase II transcription factor	0.0000
CG14422	9	12	-3.8	-2.9	RNA binding /nucleic acid binding/transcription regulator	0.0115
*Antp*	24	25	-1.5	-1.4	specific RNA polymerase II transcription factor	0.0228
**Enzymes and enzyme regulators**						
*ia2*	351	276	7.3	5.8	protein tyrosine phosphatase	0.0000
CG1827	32	26	2.3	1.8	N4-(beta-N-acetylglucosaminyl)-L-asparaginase	0.0010
*ninaC*	22	22	1.8	1.9	myosin ATPase, protein serine /threonine kinase	0.0127
G-iα65A	322	259	1.8	1.4	heterotrimeric G-protein GTPase	0.0028
Sucb	148	142	1.6	1.5	succinate-CoA ligase	0.0096
CG7288	117	125	1.5	1.6	ubiquitin-specific protease	0.0040
CG8913	50	69	1.1	1.5	peroxidase	0.0000
CG9236	7	5	-2.6	-3.4	calcium-dependent protein serine/threonine phosphatase	0.0199
**Transporters**						
CG8533	16	12	-2.6	-3.5	glutamate-gated ion channel	0.0000
CG6293	28	49	-2.5	-1.4	L-ascorbate:sodium symporter	0.0004
Atpa	105	126	-1.5	-1.3	sodium/potassium-exchanging ATPase	0.0003
Fatp	240	219	-1.4	-1.5	long-chain fatty acid transporter	0.0003
**Signal transducers**						
*usp*	41	137	8.1	27.3	transcription factor, DNA binding, ligand-dependent nuclear receptor, ecdysteroid hormone receptor	0.0001
*ninaC*	22	22	1.8	1.9	myosin ATPase, serine/threonine kinase, calmodulin binding	0.0127
CG8533	16	12	-2.6	-3.5	glutamate-gated ion channel	0.0000
**Structural protein**						
CG14889	50	50	-2.8	-2.8	extracellular matrix /structural molecule	0.0023
**Miscellaneous proteins**						
CG2807	78	105	-2.3	-1.7	pre-mRNA splicing factor	0.0034
CG32423	431	428	-2.1	-2.1	RNA binding	0.0000
XRCC1	56	20	-1.4	-3.8	DNA repair protein	0.0000
Cyp9f2	121	104	-1.5	-1.8	cytochrome P450	0.0012
Cyp9f3	77	70	-1.5	-1.7	pseudogene	0.0038
**Unknown**						
CG3857	237	186	-2.4	-3.0	NA	0.0000
CG7986	130	117	-1.5	-1.7	NA	0.0009
CG31150	66	69	-1.5	-1.4	NA	0.0160
CG33187	148	125	-1.3	-1.6	NA	0.0035

Previous studies have shown that the *Drosophila *activin signaling pathway partially mediates neuronal remodeling through regulating EcR-B1 expression [17]. Two independent mutations that block neuronal remodeling in the mushroom bodies (MBs) during pupation were found to reside in *babo *and *dSmad2 *[17], both of which have been shown to participate in the activin signaling pathway [7,9]. Further, mutations in these signaling components reduce the expression of EcR-B1, and restoration of EcR-B1 expression rescues neuronal remodeling defects. These observations led to the model that the *Drosophila *activin signaling results in induction of the EcR-B1 isoform. Upon binding of ecdysone to the EcR-B1/USP heterodimeric receptors, neuronal remodeling is initiated via transcriptional activation of downstream target genes [17]. Our microarray analysis shows that high level expression of *usp *is also induced by ectopic expression of *tkv *and *babo*. In addition, we find that EcR-A expression is repressed by the induction of *babo*. Using real-time PCR, we confirmed that EcR-B1 is induced by ectopic expression of *babo *(1.5 fold), a more modest change than the increases on *usp *by *tkv *and *babo*. These finding suggest that *Drosophila *activin signaling mediates neuronal remodeling by regulation of both EcR-B1 and *usp *expression, while inhibiting EcR-A induction.

BMP-like pathways, as well as activin pathways, have been implicated in neuronal remodeling [13,17]. PUNT and WIT have been shown to have a redundant function in inducing EcR-B1 expression during brain development. In mutant clones, levels of EcR-B1 were unaffected, unless both receptors were mutant. These results are consistent with our findings that activated *tkv *and *babo *both induce EcR-B1, although it is not known which receptor combinations or ligands are responsible for these effects.

*dpp *(and presumably *tkv*) has other known roles in organizing the visual center of the brain [13]. It has been shown that *wingless*, acting through *dpp*, is an important participant in organizing the optical centers of the brain [13]. *wingless *is expressed at the tips of the crescent shaped OPC. Fourteen hours later, *wingless *induces *dpp *expression in adjacent cells, in two spots in each brain hemisphere. These *dpp *expressing cells also express fasciclin II. BrdU staining shows that *wingless*, *dpp*, and fasciclin II expressing cells proliferate throughout larval development. However, a reduction of *wingless *or *dpp *results in a reduction in the rate of proliferation in the OPC, resulting in smaller optic lobes of the brain. Loss of *wingless *also results in a severe reduction of the medulla, where the photoreceptor axons R7 and R8 migrate. Another defect noted in *wingless *mutant animals is that the OPC derived precursor cells had failed to assume their proper neuronal fate.

### Transcription factors regulated by both DPP and activin pathways

Besides *usp*, two other transcription factor genes, CG7839 and TfIIFβ, are up regulated by *tkv *and *babo*. Both are implicated in growth processes. CG7839 has 30% homology over 1016 residues to *C. elegans *F23B12.7, which shows a slow growing phenotype in RNAi experiments [24]. TfIIFβ is part of the RNA transcriptional machinery, and 28% of glioblastomas and 80% of astrocytomas show amplification of this gene. Perhaps part of the growth potential of the *tkv *and *babo *TGFβ pathways operate through these transcription factors.

Two transcription factors, CG14422 and Antennapedia (*antp*), are down regulated by both pathways during brain development. *antp *is a well-studied Hox gene in *Drosophila*, which controls many developmental decisions, most notably, the differentiation of the antennae and legs from homologous structures [25]. The enormous diversity of body plans in animals is partially due to the variations that Hox transcription factors regulate gene expression. Most animals have one or more clusters of *Hox *genes, and each *Hox *gene controls the development of a specific region of the body plan [26]. In *Drosophila*, differences between segments, such as the presence or absence of appendages, are often controlled by Hox transcription factors. The role of *antp *in brain development is not known, but it is tempting to speculate that both *dpp *and activin might regulate brain development, at least partially, through interaction with the Hox gene *antp*. Determining the mechanisms by which Hox proteins regulate gene expression will be important for understanding animal development and pattern formation.

### Other genes regulated by *tkv *and *babo *pathways

Many of the other genes that are significantly regulated by *tkv *and *babo *are evolutionarily conserved throughout animal phyla. Quantitative analysis of transcript levels indicates that TGFβ controls some genes that encode kinases and phosphatases that might be involved in signaling pathways. For example, *ia2*, a transmembrane receptor protein phosphatase [27], has the highest level of transcriptional change among these kinases and phosphatases. Antibodies to the human version of the gene are often indicative of diabetes [28-30]. *NinaC *is a protein serine/threonine kinase [31] with calmodulin binding activity [32]. CG9236 is a calcium-dependent protein serine-threonine phosphatase, which is down regulated. It is strongly related to *C. elegans *F30A10.1, which is involved in negative regulation of body size. If the function of the protein has also been conserved, then down-regulation by the TGFβ-like pathways would allow growth in the developing brain. Other kinases and phosphatases co-regulated by both TGFβ pathways are G-iα65A (G-ialpha65A), a G-protein coupled receptor protein involved in neuroblast cell division and cell size control [33,34], and CG 9236, a calcium-dependent protein serine/threonine phosphatase [27].

CG3857 and CG7986 are two novel proteins that have homologs in *C. elegans *and in vertebrates. While their molecular functions are not currently known, the *C. elegans *CG7986 homolog F41E6.13 is involved in positive regulation of growth. RNAi experiments with the *C. elegans *homolog of CG3857, Y54E2A.2, revealed no mutant phenotype. Four transporters (Atpa, Fatp, CG8533, CG6293) are transporters regulated by the *tkv *and *babo *pathways. Fatp is a long-chain fatty acid transporter. Atpa is a sodium/potassium-exchanging ATPase, while CG8533 is a glutamate-gated ion channel and CG6293 is a L-ascorbate:sodium symporter.

## Conclusions

Microarray experiments revealed that 27 genes are co-regulated in both *tkv *and *babo *signaling pathways in the developing *Drosophila *brain. One of the most striking developmental events in the fly brain is neuronal remodeling. These results indicate *usp *is positively regulated by *tkv *and *babo*, and thus adds another important link to their roles in brain remodeling. Many of the 27 genes are strongly conserved in other species. If their biological functions are also conserved, then the RNAi experiments in their *C. elegans *counterparts show that several of them are involved in growth regulation. This is particularly useful since few downstream targets of BMP or activin signaling pathways are known, particularly the targets that execute their growth regulatory properties. Not surprisingly, mutational analysis of several of these genes has not been done, but the genetic tools in *Drosophila *make this relatively straightforward. Further characterization of these downstream genes may provide insights into the integration of *tkv *and *babo *signaling pathways in *Drosophila *brain development, and provide hints into their functions in other organisms.

## Methods

### Fly stocks

For over-expression of constitutively activated *tkv*, virgin females from UAS-CA-*tkv *were crossed to *hs*-Gal4 males. For over-expression of constitutively activated *babo *and the control, UAS-CA-*babo *and UAS-*gfp *were crossed to *hs*-Gal4 flies. The larvae were raised in standard medium at 25°C.

### Heat shock treatment and RNA purification

Wandering third-instar larvae were heat-shocked to induce ectopic expression of *tkv, babo*, and the *gfp *control (UAS-*gfp; hs*-GAL4). Animals were heat shocked at 37°C for 1 hour, followed by cooling to room temperature for 30 minutes, and then kept at 25°C for one hour to allow expression before dissection. Approximately 150-200 larvae were dissected and the brains were collected in a drop of PBT (PBS, 0.01% Tween-20, pH 7.4) on Sylgard (Dow Corning). Total RNA was extracted from the tissue using the Trizol™ reagent (Invitrogen, Carlsbad, CA) according to the manufacturer's protocol.

### Preparation of labeled cRNA

Total RNA from each of nine independent samples (three *tkv*, three *babo *and three *gfp*) was prepared for hybridization according to the Affymetrix GeneChip^® ^Expression Analysis Technical Manual (Affymetrix, Santa Clara, CA). The Superscript Choice System kit (Invitrogen, Gaithersburg, MD) was used to make complementary DNA (cDNA) from 5 μg. First strand synthesis was primed with a T7-(dT)_24_oligonucelotide primer containing a T7 RNA polymerase promoter sequence on the 5' end (Genset Oligos, La Jolla, CA). Second strand products were cleaned with the GeneChip^® ^Sample Cleanup Module (Affymetrix, Santa Clara, CA) and used as a template for *in vitro *transcription (IVT) with biotin-labeled nucleotides (Bioarray High Yield RNA Transcript Labeling Kit, Enzo Diagnostics, Farmindale, NY). The copy RNA (cRNA) product was cleaned with the GeneChip^® ^Sample Cleanup Module (Affymetrix, Santa Clara, CA) and a 20 μg aliquot was heated at 94°C for 35 min in fragmentation buffer provided with the Cleanup Module (Affymetrix, Santa Clara, CA).

### Microarray hybridization

Fifteen μg of adjusted cRNA from each sample was hybridized for 16 hr at 45°C to an Affymetrix (Santa Clara, CA) *Drosophila *Genechip 1 array. After hybridization, each array was stained with a streptavidin-phycoerythrin conjugate (Molecular Probes, Eugene, Oregon), washed and visualized with a Genearray™ Scanner (Agilent Technologies, Palo Alto, CA). Images were inspected visually for hybridization artifacts. In addition, quality assessment metrics were generated for each scanned image and evaluated based on empirical data from pervious hybridizations and on the signal intensity of internal standards that were present in the hybridization cocktail. Samples that did not pass quality assessment were eliminated from further analyses.

### Generation of expression values

Microarray Suite version 5 (Affymetrix, Santa Clara, CA) was used to generate *.cel files. Probe Profiler™ version 1.3.11 software (Corimbia Inc, Berkeley, CA) was used to convert cel file intensity data into quantitative estimates of gene expression for each probe set. For each probe set, a probability statistic is generated. Genes not significantly expressed above background in any of the samples (P > 0.05) were considered absent. Absent genes were removed from the data set and not included in further analyses.

### Data analysis

#### Tests of Significance

Gene expression levels were subjected to a 1-way analysis of variance (Anova) for 3 treatments (B, C, T) and 3 replications using AnalyzeIt Tools, a custom software program developed by the Interdisciplinary Center for Biotechnology Research (ICBR, University of Florida), for the analysis of microarray data. In this software, the statistical package, R, serves as the backend for Anova. Genes were considered to have a significant treatment effect if P-level was less than 0.05.

The expression values of those genes that were considered to have a significant treatment effect were normalized by performing a Z- transformation [35], thereby generating a distribution with mean 0 and standard deviation of 1 for each gene. Hierarchical clustering, K-Means clustering and Principal Component Analysis were performed on normalized values using GeneLinker™ Gold 3.1 (Predictive Patterns, Kingston, Ontario).

To eliminate noise from low-level expression, spots quantified less than 5 were replaced by value 5. The following criteria were used to filter the data. Only transcripts with the fold change difference over 1.5 (*tkv *(or *babo*) average/Control average or Control average/*tkv *(or *babo*) average) and statistically significant (P <= 0.05, analysis of variance (Anova)) were considered as differentially expressed. AnalyzeIt Tools and notations in Flybase were used for classification of genes by gene ontology in molecular function and biological process categories.

### Real time RT-PCR

Two independent total RNA samples were generated for each of the three experimental conditions (two *tkv*, two *babo *and two *gfp*). Each of the samples were analyzed three independent times, resulting in six repeats. These six repeats were averaged and the *tkv *and *babo *samples were compared with the *gfp *controls. Approximately 1 μg of the each total RNA was used for first strand cDNA reaction using Superscript First Strand Synthesis kit (Invitrogen, Carlsbad, CA) according to the manufacturer's protocol. For real-time PCR, the reaction consisted of cDNA first strand template, primer mix, Rox (Invitrogen, Carlsbad, CA) and SYBR Green PCR Master Mix (Invitrogen, Carlsbad, CA) in a total volume of 25 μl. Three reactions per template were performed in parallel. Actin 42F was used as an internal standard to generate a standard curve and to normalize the amount of cDNA samples. The fold change (as presented in Fig 2) was calculated from the average real time PCR data: (*tkv *or *babo*) average/Control average or Control average/(*tkv *or *babo*) average. The experiments were performed using a Rotor Gene 3000 (Corbett Research, Sydney, Australia). To validate the specificity of PCR reaction, a melting curve was produced by denaturation of PCR end products from 60 to 99°C at 0.5°C/min steep and the end products were also assayed with 1.5% agarose gel electrophoresis after cycling.

## Authors' contributions

MY and DN carried out experiments in the project and YF assisted in the experimental design. RWP implemented and supervised the project. RWP and MY prepared the manuscript.
